# Lateral Meniscus Hypermobility: Current Concepts in Pathoanatomy, Diagnosis, and Evolving Treatment Strategies

**DOI:** 10.7759/cureus.93312

**Published:** 2025-09-26

**Authors:** Zubair Younis, Muhammad A Hamid, Thomas Devasia, Faliq Abdullah

**Affiliations:** 1 Orthopaedics, The Royal Wolverhampton NHS Trust, Wolverhampton, GBR; 2 Orthopaedic Surgery, University Hospitals Birmingham, Birmingham, GBR; 3 Trauma and Orthopaedics, The Royal Wolverhampton NHS Trust, Wolverhampton, GBR; 4 Trauma and Orthopaedics, University Hospital of Wales, Cardiff, GBR

**Keywords:** arthroscopy, hypermobility, lateral meniscus, meniscus repair, popliteomeniscal fascicles

## Abstract

Hypermobility of the lateral meniscus (HLM) is an under-recognised yet clinically important source of knee pain and mechanical symptoms. It differs from conventional meniscal tears in that the meniscus itself remains structurally intact, while insufficiency of stabilising structures such as the popliteomeniscal fascicles, posterior capsule, and meniscotibial ligament permits abnormal translation. The distinctive anatomy of the lateral meniscus explains its greater vulnerability to instability compared with the medial side, particularly in young and athletic individuals. Patients commonly report episodic locking, catching, or giving way, but imaging findings are often inconspicuous, making arthroscopy the diagnostic gold standard. Dynamic arthroscopic manoeuvres, including probing, aspiration, and the figure-of-four test, reliably demonstrate pathological translation. Conservative measures may provide transient relief but are rarely curative, as the pathology reflects structural instability rather than degenerative change. Contemporary surgical management emphasises meniscal preservation through repair and reinforcement of meniscocapsular attachments, using techniques such as inside-out or all-inside sutures, fascicular plication, or capsulodesis. In cases of fascicular deficiency, reconstruction with grafts or anchors has been described, while anterior horn hypermobility, though less common, can be effectively treated with outside-in fixation. Meniscectomy, once widely performed, is now reserved for irreparable cases given its strong association with lateral compartment degeneration. Reported outcomes following meniscus-preserving repair are consistently favourable, with high rates of symptom resolution, return to sport, and joint preservation. Therefore, HLM should be regarded as a pathology of dynamic instability that requires careful diagnostic evaluation and a surgical approach focused on stabilisation rather than resection.

## Introduction and background

Hypermobility of the lateral meniscus (HLM) describes a pathologic, excessive translation of an otherwise structurally intact lateral meniscus, most commonly due to insufficiency of its posterior stabilisers, particularly the popliteomeniscal fascicles (PMFs), the posterior capsule, the lateral meniscotibial ligament, and, to a lesser extent, the meniscofemoral ligaments across the popliteal hiatus [1]. This complex anatomy explains why lateral meniscal stability differs from the medial side and why PMF injury can produce mechanical symptoms (locking, giving way, snapping) without conspicuous meniscal tearing [1,2].

Epidemiologically, HLM remains under-recognised, with retrospective series estimating it may account for approximately 2-3% of arthroscopies performed for mechanical knee symptoms [3]. Patients often present with lateral joint-line pain and episodic locking, including athletic cohorts in whom symptom provocation occurs with pivoting or external rotation [4,5]. Conventional MRI can be unrevealing, although secondary signs, such as widening of the popliteal hiatus, subtle PMF signal change, parameniscal fluid, or dynamic meniscal displacement, may raise suspicion [2,6]. Intra-articular assessment, therefore, remains pivotal; arthroscopic probing and aspiration tests, performed in valgus and “figure-of-four” positions, can demonstrate >50% translation of the posterolateral meniscus beyond the lateral tibial plateau or femoral condyle, an accepted hallmark of instability [1,7]. Additional signs, such as the lateral-gutter “drive-through,” may indicate associated posterolateral pathology that coexists with PMF injury [8].

Although most reports emphasise posterior horn hypermobility, anterior horn involvement also occurs and may present with anterolateral pain and parameniscal fluid while appearing structurally intact on MRI. Stabilisation of the anterior horn with outside-in sutures has shown favourable short-term outcomes [7]. Across the broader HLM literature, contemporary management trends have shifted decisively towards meniscus-preserving repair of the injured stabilisers via all-inside, inside-out, or posterior trans-septal techniques, giving consistent symptom relief and return to sport rates, while strategies such as thermal shrinkage are now of mainly historical interest [5,9].

Clinically, HLM is significant because it can impair knee stability, disrupt athletic performance, and lead to progressive joint degeneration if unrecognised, thereby affecting both function and quality of life. HLM is a pathology of dynamic stability rather than a purely morphologic one. A high index of suspicion, careful scrutiny of the popliteal hiatus/PMFs on imaging, and a systematic arthroscopic evaluation using dynamic tests and posterior-compartment views are essential to confirm the diagnosis and guide stabilising repair that preserves meniscal tissue [1]. In this narrative review, we summarise current concepts in the anatomy, biomechanics, pathoanatomy, clinical presentation, diagnostic evaluation, and evolving management strategies of hypermobile lateral meniscus.

## Review

Anatomy and biomechanics

Anatomy

The lateral meniscus is more mobile than its medial counterpart because of its distinctive posterolateral anatomy. Unlike the medial meniscus, which is tethered by multiple capsular and ligamentous stabilisers, the lateral meniscus has a relatively sparse attachment at the popliteal hiatus [1]. This region is traversed by the popliteal hiatus, a bare area through which the popliteus tendon passes from the intra-articular to extra-articular space [1]. The key stabilising structures include the PMFs, posterior capsule, posterior meniscotibial ligament (MTL), and meniscofemoral ligaments (MFLs: Humphrey and Wrisberg), all of which integrate with the popliteus tendon complex [10].

The PMFs consist of three variably present fascicles, namely, anterior, posterosuperior, and posteroinferior. Together, these fascicles form a hoop-like sling around the popliteus tendon and function as critical restraints against meniscal translation. This sling configuration is functionally significant because it allows the PMFs to tighten dynamically during knee flexion and rotation, synchronising meniscal stability with popliteus activity and thereby preventing pathological anterior translation of the lateral meniscus. They link the lateral meniscus to the popliteus tendon and musculotendinous unit, acting as dynamic restraints during knee motion [1,9]. The MFLs, originating from the femoral condyle and inserting into the posterior horn of the lateral meniscus, function as secondary stabilisers and may antagonise the posterior pull of the popliteus unit [11]. The posterior meniscotibial ligament (MTL) anchors the lateral meniscus to the tibial plateau, running parallel to the posterior capsule. It provides resistance to excessive posterolateral translation and is thought to be a key restraint in hyperextension [11]. Anatomical variants, such as congenital absence of the PMFs or MTL (Wrisberg variant of discoid meniscus), predispose to hypermobility even in the absence of trauma [1].

Although most descriptions focus on the posterior horn, hypermobility may also affect the anterior horn of the lateral meniscus. In this setting, the anterior horn can translate abnormally across the tibial plateau, a phenomenon that is rarely reported but clinically significant [7].

Biomechanics

The lateral meniscus functions primarily as a load distributor within the knee, transmitting and dispersing axial loads across the tibiofemoral joint to reduce peak contact stresses on the articular cartilage [1]. In doing so, it also serves as an effective shock absorber, protecting cartilage and subchondral bone from high-impact forces. Beyond load distribution, the lateral meniscus provides secondary stabilisation, resisting excessive translation during complex joint motion. Its stabilising role is highly dependent on the integrity of its fascicular and capsular attachments.

Biomechanical studies have demonstrated that the PMFs are particularly important in restraining anterior translation of the posterior horn, especially under flexion and rotational stresses. Simonian et al. showed that sequential sectioning of the aPMF and psPMF increased anterior lateral meniscus translation by nearly 80% under simulated anterior tibial loading at 90° of knee flexion, highlighting their critical contribution to meniscal stability [12]. This effect becomes more pronounced during flexion combined with external rotation, when hoop stresses are critical for maintaining meniscal congruency with the femoral condyle. Lesions of a single fascicle may not suffice to cause hypermobility, but combined injuries, particularly involving both the PMFs and posterior MTL, can disrupt hoop stresses and produce clinically relevant instability [13]. Given that the lateral meniscus normally bears up to 70% of the compartmental load in flexion and contributes to secondary restraint against anterior translation, loss of fascicular integrity places the joint at risk of abnormal contact mechanics, pain, and accelerated cartilage wear.

The MFLs counteract the posterior and lateral pull of the popliteus and PMFs by exerting an anterior-medial force on the meniscus during knee flexion and rotation. In situations where the PCL or posterior meniscal root is deficient, the MFLs assume greater responsibility in maintaining anteroposterior and rotational stability [11].

The posterior MTL and capsule limit hyperextension and resist posterolateral rotation. While their biomechanical role has been less extensively studied than the PMFs, their insufficiency has been implicated in lateral meniscal hypermobility [14].

Dynamic contributions also arise from the popliteus musculotendinous unit, which controls external rotation and varus stress, and prevents intra-articular entrapment of the meniscus between femoral and tibial surfaces during motion [12].

Lateral meniscus stability results from a complex interplay between fascicular, capsular, and ligamentous structures. Isolated disruptions may have modest effects, but cumulative injuries compromise hoop stresses and predispose to symptomatic hypermobility. This unique anatomy explains why the LM is more vulnerable than the medial meniscus and underscores the importance of preserving stabilising structures during surgery [12,13].

Pathoanatomy, epidemiology, and risk factors

Pathoanatomy

HLM is fundamentally a disorder of stabilising structures rather than the meniscus substance itself. The posterior horn of the lateral meniscus is most commonly affected, reflecting the importance of the PMFs, posterior capsule, and posterior MTL in restraining abnormal excursion [1,9]. Disruption or attenuation of the PMFs, whether traumatic or degenerative, leads to excessive anteroposterior translation of the lateral meniscus [11]. Tears may occur in isolation but often coexist with posterolateral corner (PLC) injuries, popliteus tendon avulsions, or meniscal root lesions [10].

Arthroscopically, instability is demonstrated by anterior translation of the posterior horn beyond the lateral femoral condyle or tibial plateau under valgus and external rotation stress [11]. In some cases, a hypermobile anterior horn has also been described, manifesting as forward subluxation across the tibial plateau, despite intact meniscal morphology [6]. If left untreated, repetitive subluxation can induce meniscocapsular scarring, parameniscal cyst formation, and eventually cartilage wear of the lateral compartment [9]. Experimental studies highlight the quantitative stabilising role of the PMFs under different loading conditions (Table 1).

**Table 1:** Comparative biomechanical evidence on PMF function. PMF: popliteomeniscal fascicle; LM: lateral meniscus

Study	Sample size (knees)	Test conditions	Key findings
Simonian et al [12].	12 cadaveric knees	90° flexion with anterior tibial load	Sectioning aPMF + psPMF ↑ anterior LM translation by ~80%
LaPrade et al. [11].	10 cadaveric knees	Flexion + external rotation	PMF sectioning increased lateral compartment gapping, especially under varus stress
Keyhani et al [13].	14 patients (arthroscopic + MRI correlation)	Dynamic probing in the figure-of-four	Posterior trans-septal repair restored stability comparable to the intact state

Epidemiology

The true incidence of HLM remains uncertain due to underdiagnosis and the absence of specific MRI criteria [2]. Retrospective series have suggested that HLM accounts for a minority of symptomatic lateral meniscus cases, yet is disproportionately represented among young and athletic populations [4]. In one surgical cohort of soccer players, meniscal fixation for HLM yielded symptom resolution and high return-to-sport rates, indicating a clinically relevant burden in athletes [5].

Case reports also highlight HLM in adolescents and young adults presenting with unexplained locking or giving way, where standard imaging appears normal [9]. In contrast, degenerative or atraumatic HLM is rare but can arise in older patients, often in association with capsular laxity or discoid meniscus variants [15].

Risk Factors

Several anatomical and clinical risk factors predispose an individual to HLM. These factors can be broadly categorised into congenital variants, traumatic events, and repetitive stress conditions. Congenital anatomical variations, such as the absence of the PMFs, a hypoplastic posterior MTL, or a Wrisberg-type discoid meniscus, which lacks a posterior tibial attachment, create a native structural vulnerability that significantly increases susceptibility to HLM [1,11,13]. Acute trauma, particularly injuries to the PMFs or the posterolateral joint capsule, is another major risk factor; this is especially true when these injuries occur in conjunction with PLC disruptions, as they can directly destabilise the meniscus [11].

Beyond single-event trauma, repetitive athletic loading from high-demand sports that involve frequent pivoting, deep squatting, and forceful external rotation of the knee can gradually place excessive stress on the popliteomeniscal fascicular complex, leading to overstretching and predisposing the meniscus to hypermobility [5]. Furthermore, deficiency in other major stabilisers of the knee, namely, the PCL or a meniscal root tear, may shift unprecedented stabilising demands onto the MFLs, which can render the lateral meniscus vulnerable if its primary fascicular supports are already compromised [16]. Though less frequently reported, generalised joint laxity or acquired capsular insufficiency can also serve as systemic contributing factors to the development of symptomatic hypermobility [10,15]. In summary, these risk factors illustrate that HLM is not a random pathology but rather one that arises from a combination of inherent anatomic vulnerability, mechanical overload, and traumatic disruption of the knee's critical posterolateral stabilising elements.

Importantly, these risk factors may interact cumulatively. For example, a congenital variant such as absent PMFs establishes a structurally vulnerable baseline that can be further destabilised by repetitive athletic stresses, hastening fascicular attenuation and insufficiency. Likewise, traumatic injury in the presence of pre-existing ligamentous laxity or a Wrisberg-type discoid meniscus may accelerate the progression from subclinical hypermobility to symptomatic pathology. Thus, HLM development can often be understood not as the result of a single insult but as the compounded effect of overlapping vulnerabilities and stressors acting on the posterolateral stabilising complex.

Clinical presentation

Patients with HLM typically present with mechanical symptoms in the absence of obvious meniscal tears. The most frequently reported complaints are episodic locking, catching, giving way, and lateral joint-line pain, often exacerbated by pivoting, squatting, or sports-specific manoeuvres [1,10]. Symptoms may mimic those of a meniscal tear but are unique in their dynamic and intermittent nature, reflecting the abnormal translation of an otherwise intact meniscus [17].

On physical examination, tenderness along the lateral joint line is common, while provocative manoeuvres such as McMurray’s test may reproduce snapping or pain. However, standard meniscal tests lack sensitivity and specificity for HLM [12]. In athletes, complaints are often activity-related, and some describe a “snapping” sensation during external rotation and valgus stress [4,5].

While the posterior horn is the usual site of pathology, anterior horn hypermobility may cause anterolateral knee pain with a sensation of shifting or instability during extension, despite normal imaging [7]. Additionally, LaPrade et al. described the “figure-of-four test,” in which placing the knee in flexion, varus, and external rotation may reproduce lateral joint line pain due to medial displacement of the lateral meniscus when the popliteomeniscal fascicles are ruptured. This test has been reported as a useful adjunct in raising suspicion for HLM, though its validation is limited to small case series [1].

Diagnosis

Imaging

Standard MRI findings in HLM are often inconspicuous, making diagnosis challenging [2]. Indirect signs include widening of the popliteal hiatus, PMF signal irregularity, parameniscal fluid collections, and dynamic meniscal displacement [6]. Some studies suggest that a widened popliteal hiatus (>1.5 mm) is associated with meniscal instability [18]. Dynamic MRI sequences, though less widely available, may enhance detection [19]. Dynamic MRI, which images the knee under valgus and external rotation stress, can directly capture pathological anterior translation of the meniscus, confirming the diagnosis. However, its use is limited by availability and a lack of standardisation.

Arthroscopy

Arthroscopy remains the gold standard for the definitive diagnosis of HLM. The intraoperative evaluation is a dynamic process that involves a careful assessment of meniscal mobility. With the knee positioned in valgus and the classic “figure-of-four” position, which places stress on the posterolateral structures, the surgeon uses a probe to manipulate the posterior horn of the lateral meniscus. A diagnosis of instability is confirmed when more than 50% of the meniscal body can be translated anteriorly beyond the margin of the lateral femoral condyle or the tibial plateau [1,8,12].

Several specific arthroscopic tests are employed to assess the integrity of the stabilising structures. The aspiration test identifies instability by demonstrating increased anterior translation of the posterior horn when negative intra-articular pressure is created, removing the suction effect that normally helps seat the meniscus [19]. In practice, this is most reliably performed at approximately 30° of knee flexion with light probe pressure applied to the posterior horn. The lateral gutter drive-through sign is another key manoeuvre; if the arthroscope can be easily passed between the lateral meniscus and the femoral condyle into the lateral gutter, it indicates a significant insufficiency of the posterolateral capsule or the PMFs [8]. Furthermore, the figure-of-four manoeuvre itself is not just a positioning tool but also a diagnostic test, as it accentuates any existing posterior horn subluxation by tightening the posterolateral soft-tissue structures [1]. The figure-of-four manoeuvre is typically performed with the knee flexed to about 20° and a mild valgus stress applied, which may reproduce lateral joint-line pain or visible meniscal excursion.

During arthroscopy, direct visualisation often reveals a meniscus that is morphologically intact but exhibits abnormal excursion. Rather than a frank tear, the surgeon may instead observe associated lesions that explain the instability, such as tears of the PMFs, a separation at the meniscocapsular junction, or injuries to the popliteus tendon [4,12].

Differential diagnosis

The clinical picture of HLM may be confused with other intra-articular pathologies, including discoid meniscus, lateral meniscal tears, or loose bodies. Distinguishing features include normal meniscal morphology with excessive excursion, normal or subtle MRI findings, and confirmation with arthroscopic probing [2].

Management

Conservative Management

Conservative treatment for HLM is of limited utility. Unlike degenerative meniscal lesions, the underlying pathology in HLM is instability due to fascicular or capsular insufficiency rather than a structural tear that can remodel with rest [1,10]. Initial non-operative measures such as rest, physiotherapy focused on strengthening periarticular muscles, proprioceptive training, and anti-inflammatory medications may provide transient symptom relief [3]. However, repeated locking, painful snapping, or recurrent giving-way episodes are highly predictive of persistent symptoms that necessitate surgical intervention [4].

In children or adolescents with mild, intermittent symptoms, short-term conservative monitoring may be justified. Yet, prolonged delays risk progressive meniscal instability, parameniscal scarring, and secondary chondral damage [10]. Importantly, even during conservative phases, a multidisciplinary approach that includes orthopaedic surgeons, physical therapists, and sports medicine specialists is recommended to optimise rehabilitation, address biomechanical risk factors, and guide return-to-activity decisions.

Surgical Management

Principles: The contemporary surgical principle is meniscal preservation and restoration of stability. Historically, subtotal or total meniscectomy was performed, often yielding immediate symptom relief. However, long-term studies have shown accelerated lateral compartment degeneration and increased risk of osteoarthritis, making meniscectomy a last resort [1,14].

Operative options for HLM aim to restore stability by reinforcing the meniscocapsular junction, repairing or reconstructing deficient PMFs, and, in selected cases, securing the meniscus directly to the tibial plateau or capsule. Meniscectomy is reserved only for irreparable pathology [1,10]. A coordinated multidisciplinary pathway involving surgeons, physiotherapists, and, when needed, pain specialists or rehabilitation experts, is critical to maximise surgical outcomes and reduce recurrence.

Meniscal Repair Techniques

Inside-out repair: Traditionally regarded as the gold standard for posterolateral meniscal repair, this technique involves passing sutures from inside the joint to outside the capsule, retrieved through a lateral incision. It offers strong fixation but carries the risk of neurovascular injury if not carefully performed [20].

Outside-in repair: This technique allows safe fixation of anterior and middle horn lesions and can be adapted for anterior horn hypermobility. Sutures are passed from outside the capsule into the joint and tied externally.

All-inside repair: Newer devices allow fixation without accessory incisions, reducing operative time and risk to adjacent neurovascular structures. Biomechanical testing has shown comparable stability to inside-out methods for posterior horn lesions, although cost and implant-specific complications remain considerations [21].

Posterior trans-septal portal repair: Particularly useful for lesions involving the posteroinferior PMF, this approach provides direct access for secure repair of difficult-to-reach fascicular injuries [13].

Reconstruction of PMFs: When the fascicles are completely deficient or attenuated, reconstruction using hamstring autograft, tibial anchors, or capsular advancement techniques has been described. Early series report significant improvement in stability and function with low recurrence rates [9].

Adjunctive and Alternative Procedures

In addition to standard repair techniques, several adjunctive procedures have been described for managing hypermobile lateral meniscus. Thermal capsulorrhaphy, performed with arthroscopic radiofrequency shrinkage of capsular tissue, has been proposed in cases of fascicular laxity. While short-term symptom improvement has been reported, its use has declined because of concerns regarding capsular weakening and high recurrence rates [22].

Anchor-based fixation techniques represent another option, whereby tibial anchors or suture devices are used to secure the posterior horn to the tibial plateau. By restoring hoop tension, this approach provides additional stability, particularly in athletic patients, and has been associated with high return-to-sport rates [5].

For cases involving the anterior horn, arthroscopic stabilisation by suturing the meniscus to the capsule has shown promising results. This technique effectively resolves instability and pain while preserving meniscal tissue, with recent case series demonstrating excellent clinical outcomes [7].

Although short- to mid-term results of HLM repair are generally favourable, systematic reviews report limited data on complication rates, with most studies being small case series [10]. Reported complications include recurrent instability, persistent pain, or implant-related issues, but exact rates remain underexplored.

Outcomes

The literature consistently supports repair over meniscectomy in the management of hypermobile lateral meniscus. Systematic reviews demonstrate high rates of symptom resolution and functional recovery following repair of the PMFs or capsule [10]. A recent systematic review of 20 studies (212 patients) found that arthroscopic repair, particularly all-inside and inside-out techniques, was the predominant and successful treatment, leading to significant improvements in functional scores such as the Lysholm and International Knee Documentation Committee [10].

In athletic populations, outcomes are particularly favourable, with more than 80% of patients returning to their previous level of sport within four to six months after repair [4,5]. However, a large case series of soccer players highlighted a key distinction: while 82% returned to sport, only 56% returned to their preinjury activity level, underscoring the challenge of achieving peak performance post-repair [4]. The average follow-up period across studies was 29.5 months, ranging from short-term (four months) to long-term (73 months), though the evidence is primarily derived from retrospective case series (Level IV), indicating a need for higher-quality prospective studies [10]. This reinforces the importance of meniscal preservation strategies in high-demand individuals.

Recurrence of instability after repair is considered rare when fascicular integrity is adequately restored. Failures, when they occur, are often attributed to incomplete repair or unrecognised associated injuries to the posterolateral capsule [9]. In the long term, meniscectomy remains associated with accelerated lateral compartment degeneration and a significantly higher risk of osteoarthritis, underscoring its role as a salvage option only in irreparable cases [1,14].

More recently, reports of anterior horn hypermobility have shown that fixation techniques yield similarly encouraging short-term results, effectively resolving pain and instability, although long-term evidence in this subgroup is still limited [7].

## Conclusions

HLM is an increasingly recognised cause of mechanical knee symptoms, often overlooked due to its subtle imaging findings and absence of frank meniscal tearing. A detailed understanding of the complex anatomy and biomechanics of the lateral meniscus, particularly the role of the PMFs and associated stabilising structures, is essential for accurate diagnosis and effective treatment. Careful clinical evaluation combined with arthroscopic probing remains the cornerstone of diagnosis, as conventional MRI may fail to detect this dynamic pathology. Management has shifted decisively towards meniscus-preserving approaches that restore stability while maintaining tissue integrity. Techniques such as meniscocapsular repair, fascicular reinforcement, and capsulodesis have shown excellent outcomes, while meniscectomy is reserved only for irreparable cases. Anterior horn hypermobility, though less common, can also be successfully addressed with targeted repair strategies. Future efforts should focus on refining imaging modalities for earlier recognition, standardising diagnostic arthroscopic tests, and generating long-term outcome data on newer repair and reconstruction techniques. By preserving meniscal function and restoring stability, current treatment strategies offer patients not only symptom relief and return to activity but also the potential for durable joint protection.
